# Survival of bladder or renal cancer in patients with *CHEK2* mutations

**DOI:** 10.1371/journal.pone.0257132

**Published:** 2021-09-09

**Authors:** Elżbieta Złowocka-Perłowska, Tadeusz Dębniak, Marcin Słojewski, Thierry van de Wetering, Aleksandra Tołoczko-Grabarek, Cezary Cybulski, Rodney J. Scott, Jan Lubiński

**Affiliations:** 1 Department of Genetics and Pathology, International Hereditary Cancer Center, Pomeranian Medical University, Szczecin, Poland; 2 Department of Urology and Oncological Urology Clinic, Pomeranian Medical University, Szczecin, Poland; 3 Department of Clinical and Molecular Biochemistry, Pomeranian Medical University, Szczecin, Poland; 4 School of Biomedical Sciences & Pharmacy, Centre for Information-Based Medicine, Hunter Medical Research Institute, University of Newcastle, New Lambton Heights, Newcastle, NSW, Australia; 5 Division of Molecular Medicine, Pathology North, NSW Pathology, Newcastle, NSW, Australia; CNR, ITALY

## Abstract

**Purpose:**

The purpose of this study was to compare the clinical characteristics and the survival of *CHEK2* mutation positive and *CHEK2* mutation negative patients diagnosed with bladder or kidney cancer.

**Materials and methods:**

1016 patients with bladder and 402 cases with kidney cancer and 8302 controls were genotyped for four *CHEK2* variants: 1100delC, del5395, IVS2+1G>A and I157T. Predictors of survival were determined among *CHEK2* pathogenic variant carriers using the Cox proportional hazards model. The median follow-up was 17.5 years. Covariates included age (≤60; >61 years), sex (female; male), clinical characteristics (stage: TNM, grade, histopathological type), smoking status (non-smoking; smoking) and cancer family history (negative; positive).

**Results:**

We found no impact of *CHEK2* mutations on bladder or kidney cancer survival. However, we observed a possible increased survival in the subgroup of patients with stage T1 bladder cancer with *CHEK2* mutations but this did not meet statistical significance (HR = 0.14; 95% CI 0.02–1.04; p = 0.055). Moreover, we observed that the missense mutations were more frequent in the low grade invasive bladder cancer patient group (OR = 7.9; 95% CI 1.50–42.1; p = 0.04) and in patients with bladder cancer with stage Ta (OR = 2.4; 95% CI 1.30–4.55; p = 0.006). The different results where missense mutations occurs less often we observed among patients with high grade invasive bladder cancer (OR = 0.12; 95% CI 0.02–0.66; p = 0.04) and those with stage T1 disease (OR = 0.2; 95% CI 0.07–0.76; p = 0.01). Our investigations revealed that any mutation in *CHEK2* occurs more often among patients with stage Ta bladder cancer (OR = 2.0; 95% CI 1.19–3.47; p = 0.01) and less often in patients with stage T1 disease (OR = 0.31; 95% CI 0.12–0.78; p = 0.01). In the kidney cancer patients, truncating mutations were present more often in the group with clear cell carcinoma GII (OR = 8.0; 95% CI 0.95–67.7; p = 0.05). The 10-year survival for all *CHEK2* mutation carriers with bladder cancer was 33% and for non-carriers 11% (*p* = 0.15). The 10-year survival for *CHEK2* mutation carriers with kidney cancer 34% and for non-carriers 20% (*p* = 0.5).

**Conclusion:**

*CHEK2* mutations were not associated with any change in bladder or kidney cancer survival regardless of their age, sex, smoking status and family history. We observed a potentially protective effect of *CHEK2* mutations on survival for patients with stage T1 bladder cancer. *CHEK2* missense mutations were more common among patients with low grade invasive bladder cancer and in patients with stage Ta diease. The frequencies of the I157T *CHEK2* pathogenic variant were less in patients with high grade invasive bladder cancer and those with stage T1 disease. Among patients with bladder cancer with stage Ta disease, the OR for any mutation in *CHEK2* was 2.0 but for those with stage T1 disease, the OR was 0.3. We observed truncating *CHEK2* mutations were associated with kidney cancer patients with GII clear cell carcinoma.

## Introduction

Mutations in the cell cycle checkpoint kinase 2 (*CHEK2*) tumor suppressor gene are associated with multi-organ cancer susceptibility including cancers of the breast, prostate, bladder, kidney, thyroid, stomach and colon [1–11]. Three recurrent truncating mutations (1100delC, del5395, IVS2+1G>A) and one common missense mutation (I157T) in *CHEK2* gene have been found in 1.0% (both c.1100delC and c.5395del) and 4.9% of the Polish population, respectively [1, 3]. In 2004, we investigated 172 patients with bladder cancer and 264 with kidney cancer. We showed that frequency of the missense variant was significantly increased among cases with kidney cancer (9.8%; odds ratio OR 2.1; *p* = 0.0006) [1]. In 2008, we studied 416 unselected cases of urothelial bladder cancer for *CHEK2* mutations which revealed a frequency of 10.6% (OR 1.9; *p* = 0.0003) [4]. Recently we genotyped 835 patients with invasive renal cancer and 8302 adult controls. The missense mutation was present in 78 participants with renal cancer and 410 controls (9.3%; OR 2.0; *p* <0.001). *CHEK2* truncating mutations were present in 20 patients and 80 controls (2.4%; OR, 2.5; *p* = 0.0003) [2]. To validate and extend our earlier findings we evaluated the prevalence of four commons *CHEK2* mutations among 1016 patients with bladder cancer. Additionally, the goal of the current study was to evaluate the impact of these mutations on survival from the 1016 bladder cancer patients and 402 kidney cancer patients. To our knowledge, this is the first large-scale study to describe the clinical characteristics and survival of patients with bladder and kidney cancer carrying mutations in *CHEK2*.

## Material and methods

### Study population

#### Patients

**Fig 1:**
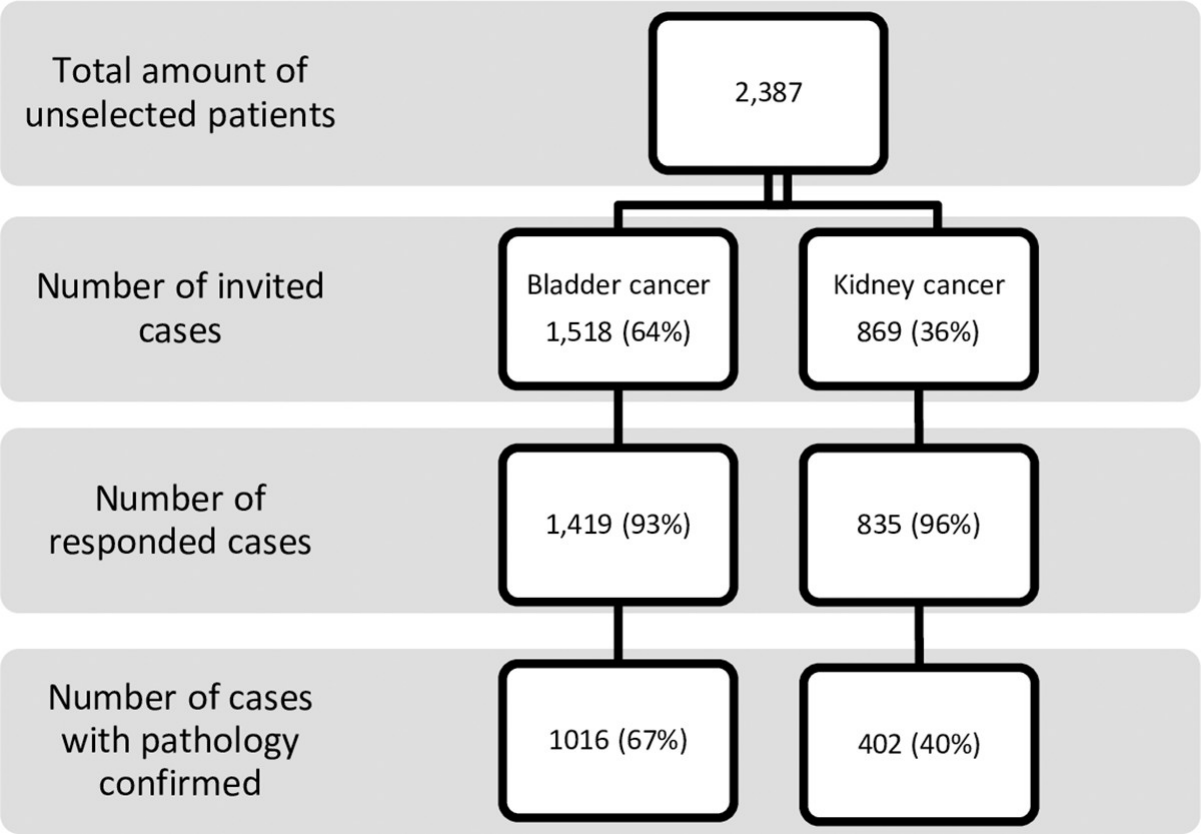
Diagram with the group included in the study.

This study comprised 1016 unselected cases of urothelial bladder cancer (233 women and 783 men) and 402 unselected kidney cancer patients (148 women and 254 men) treated at the Urology Hospital in Szczecin and the Genetic outpatients Clinic between 1986 and 2018. All patients and control subjects are of European ancestry and are ethnic Poles. A total of 1518 incident cases of bladder cancer and 869 kidney cancer were identified during the study period. Of these, 1419 patients with bladder and 835 with kidney cancer accepted the invitation to participate in the study. During the interview at the Genetic outpatients Clinic the goals of the study were explained, informed consent was obtained, family history and smoking status were collected, genetic counseling was given and a blood sample taken for DNA analysis. The pathological diagnosis of bladder and kidney cancer was confirmed by biopsy review at a single central pathology laboratory in Szczecin, Poland. All cases were unselected for age, sex, clinical characteristics (stage: T, grade, histopathological type of cancer), smoking status and family history. Clinical data were collected from the patients’ records. If information was missing on stage, grade, histopathological type of cancer then the case was excluded. Of the 1419 patients with bladder and 835 with kidney cancer, clinical information was missing for 403 bladder and 433 kidney cases and these subjects were excluded from the study. Only those patients for whom mutation status was available for all four *CHEK2* mutations were included in the study. In total, we recorded data from 1016 patients with bladder and 402 with kidney cancer, Fig 1. The mean age of diagnosis for bladder cancer patients was 67 years (range 25–91) and 64 (range 21–85) for kidney cancer patients. Detailed information of smoking status was available for a subset of 811 (80%) cases with bladder and 249 (62%) kidney cancer patients (pack years). The family tree was constructed on the basis of the family history which was established using a standardized questionnaire answered by the patient and verified during the interview with the physician. A total of 33 patients with a family history of at least 1 bladder cancer in their first or second degree relatives and 11 cases with a family history of at least 1 kidney cancer in first or second degree relatives were identified. The vital status and the date of death of all of the cases were requested from the Polish Ministry of the Interior and Administration in January 2021, which was obtained in February 2021. In total we received information that 637 (63%) patients with bladder and 126 (38%) with kidney cancer had passed away. The study was approved by the Ethics Committee of Pomeranian Medical University in Szczecin.

### Controls

The control group comprised 8302 cancer-free, population-based, adults from (the same genetically homogeneous population as the patients) Poland. In order to estimate the frequency of the *CHEK2* Polish founder mutations in the general population, two control groups were combined. The first group included 3,956 cancer-free men within the age range of 23–90 years old (mean age 61.2 years) unselected for family history. The second group consisted of 4,346 cancer-free females aged 19–91 years (mean age 52.2 years) unselected for family history. These controls are described in detail elsewhere, male controls [12] and female controls [13]. The allele frequencies for all variants in our control group were not dependent on age or sex, and the prevalence estimates of mutations in all genes were similar in younger and in older controls.

## Methods

DNA was isolated from 5 to 10 mL of peripheral blood. The three mutations in *CHEK2* (1100delC, IVS2+1G>A and I157T) were genotyped as described previously [1]. In brief, these variants are detected by ASO‐ or RFLP‐PCR analyses. The third truncating mutations in *CHEK* del5395 were genotyped as described previously and was detected by a multiplex PCR reaction [3]. In all reaction sets, positive and negative controls (without DNA) were used. All PCR reactions or enzymatic digestions were performed under a layer of mineral oil. Duplicate genotyping for quality control was performed for 382 randomly selected individuals, but no discrepancies with the initial results were found. As a further check, all mutation‐positive cases were confirmed by sequencing, with no discrepancies.

## Statistical analysis

### Survival analysis

For the survival analysis, the patients were followed from the date of diagnosis of bladder or kidney cancer until date of death or February 2021. Death was established by linkage to the Polish Vital statistics registry. Subjects in the study were linked to the records of the vital statistics Poland using a unique eleven digit identification number (PESEL). Death was all-cause mortality because the specific cause of death was not available. The median follow-up was 210 months.

Kaplan-Meier survival curves were constructed for the mutation-positive and -negative sub-cohorts. Comparison of survival curves was performed by log-rank test. A multivariable Cox regression analysis was performed on these patients. Covariates included age (≤60; >61 years), sex (females; males), clinical characteristics (stage: T, grade, histopathological type of cancer), smoking status (non-smoking; smoking) and cancer family history (negative; positive).

The survival analysis was first performed using all subjects and then on the subgroups of individuals divided according to: age, sex, clinical characteristics, smoking status and cancer family history. The effect of carrying a *CHEK2* mutation was modeled first for all mutations and then separately for missense and truncating mutations.

### Power calculation

Our large control sample size provided good statistical power for the case-control analysis. At a power level of 80% and a two-sided confidence of 0.95, the minimal number of cancer patients is 242 and 729 for kidney and bladder cancer groups, respectively. Subdividing the case and control groups into age, sex, clinical characteristics, smoking status and cancer family history, the necessary number of subjects in both groups with the same statistical power would be much higher and even this would not be technical and financial possible to perform.

### Odds ratios

The prevalence of each of the four *CHEK2* alleles was compared in bladder cancer cases and in controls. The three protein truncating mutations were studied separately from the missense variant. Odds ratios were generated from two‐by‐two tables and statistical significance was assessed using the Fisher exact test where appropriate. The odds ratios were used as estimates of relative risk and additionally were adjusted for age, sex, clinical characteristics, smoking status and cancer family history by multiple logistic regression.

#### Ethical statement

The study performed in accordance with the principles of the Declaration of Helsinki. All patients and controls provided written informed consent.

## Results

### Bladder cancer

Of the 1016 bladder patients enrolled in the study, the characteristics of the study population of bladder cancer are shown in Table 1, 79 (7.8%) carried a *CHEK2* mutation (all variants combined) (OR = 1.3; 95% CI 1.05–1.72; *p* = 0.02), including 17 (1.7%) cases with a truncating mutation (OR = 1.7; 95% CI, 1.03–2.96; *p* = 0.05) and 62 (6.1%) patients with the missense mutation (OR = 1.2; 95% CI, 0.95–1.64; *p* = 0.13). The characteristics of the patients with and without mutations are presented in Table 2.

**Table 1:** Characteristics of the study population of bladder cancer (n = 1016).

Sex	
Male	783
Female	233
Age, mean (range)	67 (25–91)
≤60	295
>61	721
Smoking status	
Yes	691 (68%)
No	123 (12%)
Missing	202
Histological features	
Noninvasive papillary	626 (62%)
Low grade^1^	329 (32%)
High grade^2^	297 (29%)
Invasive	390 (38%)
Low	7 (1%)
High	383 (38%)
Stage	
Ta	626 (61%)
T1	174 (17%)
T2	117 (12%)
T3	63 (6%)
T4	36 (4%)
Vital status	
Alived	379 (37%)
Dead	637 (63%)

^1^low grade–GI.

^2^ high grade—GII and GII.

**Table 2:** Clinical characteristics of bladder cancers; by variant alleles of *CHEK2*.

	Patients with truncating mutations (17)	p-value*	Patients with missense mutations (62)	p-value*	Patients with *CHEK2* mutations (79)	p-value*	Patients with no mutations *in CHEK2* (937)
Age of diagnosis (yr)							
Mean	67.35		67.68		67.61		66.21
Histological features							
Noninvasive Papillary							
Low grade	6/11 (55)	0.89	23/47(49)	0.75	29/58(50)	0.82	298/568 (52)
High grade	5/11 (45)	0.89	24/47(51)	0.75	29/58(50)	0.82	270/567 (48)
Invasive							
Low grade	-		2/15 (13)	0.04	2/21 (10)	0.13	7/369 (2)
High grade	6/6 (100)	0.73	13/15(87)	0.04	19/21(90)	0.13	362/369 (98)
Stage							
Ta	11/17(64)	0.93	49/62(79)	0.006	60/79(76)	0.01	569/937 (61)
T1	2/17 (12)	0.75	3/62(5)	0.01	5/79(6)	0.01	166/937 (18)
T2	2/17 (12)	0.99	5/62 (8)	0.51	7/79 (9)	0.57	109/937 (12)
T3	2/17 (12)	0.66	3/62 (5)	0.87	5/79 (6)	0.96	58/937 (6)
T4	-		2/62 (3)	0.86	2/79 (3)	0.85	34/937 (3)

* p-values are calculated with respect to carriers of noncarriers.

The study subjects were followed from the date of diagnosis until death or February 2021 (a mean of 35 years). There were 46 deaths (58%) recorded in 79 carriers of a *CHEK2* mutation compared with 591 deaths (63%) in 937 noncarriers (HR = 0.73; 95% CI 0.37–1.45; *p* = 0.4). There were 35 deaths (57%) among 62 carriers of missense mutation, and 11 deaths (65%) among 17 carriers of three truncation mutations.

**Fig 2:**
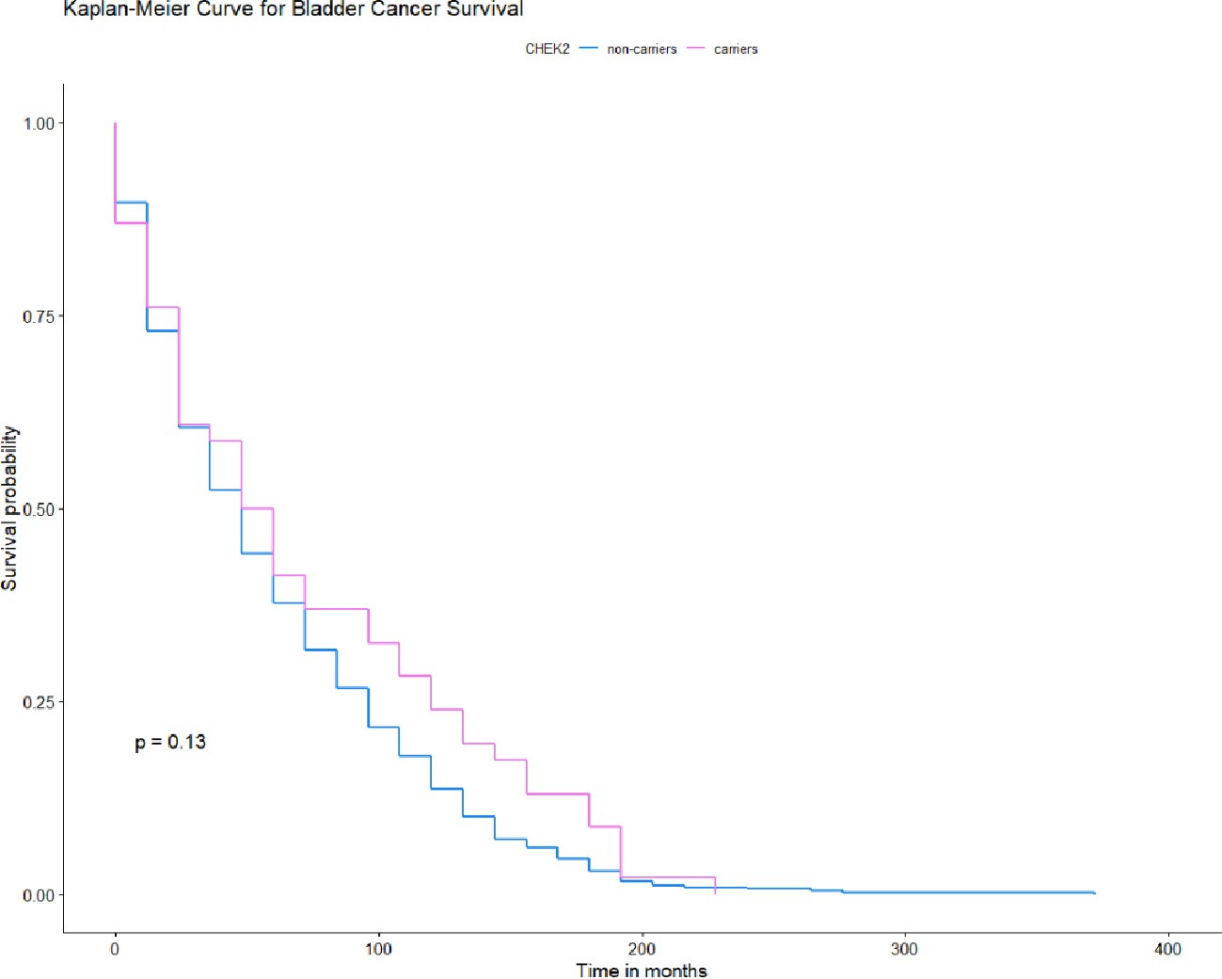
Kaplan-Meier survival curves of bladder cancer patients with any *CHEK2* mutation and -negative sub-cohorts.

None of the four *CHEK2* mutations had a significant role in the survival of the patients with bladder cancer Fig 2. We observed a possible increased survival in the subgroup of patients with stage T1 bladder cancer with *CHEK2* mutations but this was not statistical significance. The data was stratified for age, smoking status, cancer family history, sex and clinical characteristics. The median survival was 48 months for patients with a truncation mutation and 60 months for patients with the missense mutation compared to 48 months for non-carriers (Table 3). In the subgroup of patients with a truncating mutation, the 10-year survival was 24% and 15% for patients with missense mutations compared to 11% for non-carriers. After adjusting for age, smoking status, cancer family history, sex or clinical characteristics, the HR for mortality associated with bladder cancer and *CHEK2* mutation was 0.61 (95% CI 0.23–1.57; *p* = 0.3) for patients younger than 61 years old; 0.85 (95% CI 0.58–1.26; *p* = 0.4) for cases older than 61 years old; 0.73 (95% CI 0.29–1.84; *p* = 0.5) for the non-smoking group; 0.79 (95% CI 0.54–1.17; *p* = 0.2) for smoking patients; 0.79 (95% CI 0.55–1.14; *p* = 0.2) for cases with no cancer family history; 2.45 (95% CI 0.15–39.7; *p* = 0.5) for patients with positive cancer family history; 0.63 (95% CI 0.28–1.41; *p* = 0.3) for females; 0.85 (95% CI 0.57–1.26; *p* = 0.4) for males; 0.79 (95% CI 0.52–1.19; *p* = 0.3) for patients with Stage Ta; 0.14 (95% CI 0.02–1.04; *p* = 0.055) for patients with Stage T1; 1.72 (95% CI 0.67–4.42; *p* = 0.3) for patients with Stage T2; 1.31 (95% CI 0.44–3.95; *p* = 0.6) for patients with Stage T3; 5.98 (95% CI 0.65–55.3; *p* = 0.11) for patients with Stage T4; 0.79 (95% CI 0.52–1.19; *p* = 0.3) for patients with type of papillary bladder and 0.80 (95% CI 0.42–1.56; *p* = 0.5) for patients with type of invasive bladder cancer.

**Table 3:** Survival of patients with bladder cancer; by variant alleles of *CHEK2*.

	Patients with truncation mutations (n = 17)	Patients with missense mutations (n = 62)	Patients with any *CHEK2* mutation (n = 79)	Patients with no mutation in *CHEK2* (n = 937)
Median follow-up (mo)	120	108	108	96
Proportion of deceased (%)	65	57	58	63
Median survival (mo)	48	60	54	48
5-Year survival (%)	29	29	29	27
10-Year survival (%)	24	15	33	11
HR	0.87	0.75	0.78	1.0
95% CI	0.44–1.69	0.51–1.11	0.55–1.09	-
p-value	0.7	0.15	0.15	-

Hazard ratio (HR), 95% confidence interval (CI), and p-values are calculated by cox ph test. Data was stratified for age, sex, clinical characteristics, smoking status and cancer family history.

### Kidney cancer

Data on survival were available for 402 patients with kidney cancer. The characteristics of the study population of kidney cancer are shown in Table 4. The mean follow-up time was 35 years. There were 18 deaths (40%) recorded in 45 carriers of a *CHEK2* mutation compared with 109 deaths (30%) in 357 noncarriers (HR = 0.73; 95% CI 0.37–1.45; *p* = 0.4). There were 16 deaths (44%) among 36 carriers of the I157T missene mutation, and 2 deaths (22%) among 9 carriers of three truncation mutations. The characteristics of the patients with and without mutations are presented in Table 5.

**Table 4:** Characteristics of the study population of kidney cancer (n = 402).

Sex	
Male	254
Female	148
Age, mean (range)	64 (21–85)
≤60	164
>61	238
Smoking status	
Yes	142 (35%)
No	76 (19%)
Missing	184
Histological features	
Clarocellulare	
GI*	58 (14%)
GII*	157 (39%)
GIII*	99 (25%)
GIV*	28 (7%)
Chromophobe	
GI	12 (3%)
GII	2 (0.5%)
GIII	2 (0.5%)
Papillary	
GI	17 (4%)
GII	24 (6%)
GIII	3 (1%)
Stage	
Ta	4 (1%)
T1	279 (70%)
T2	35 (9%)
T3	79 (20%)
T4	5 (1%)
Vital status	Vital status
Alive	277 (69%)
Dead	125 (31%)

**Table 5:** Clinical characteristics of kidney cancers; by variant alleles of *CHEK2*.

	Patients with truncating mutations (9)	p-value*	Patients with missense mutations (36)	p-value*	Patients with *CHEK2* mutation (45)	p-value*	Patients with no mutations *in CHEK2* (357)
Age of diagnosis (yr)							
Mean	70.7		58.7		64.7		63.3
Histological							
features							
Clarocellulare							
GI	-		3/30 (10)	0.45	3/37 (8)	0.24	55/321 (17)
GII	6/7 (86)	**0.05**	14/30(47)	0.81	20/37(54)	0.25	137/321 (42)
GIII	1/7 (14)	0.74	11/30(37)	0.36	12/37(33)	0.62	87/321 (27)
GIV	-		2/30 (6)	0.78	2/37 (5)	0.79	26/321 (8)
Chromophobe							
GI	-		-		-		12/321 (4)
GII	-		-		-		2/321 (1)
GIII	-		-		-		2/321 (1)
Papillare							
GI	1/2 (50)	0.87	-		1/8 (12)	0.20	16/36 (44)
GII	1/2 (50)	1.00	5/6 (84)	0.28	6/8 (76)	0.37	18/36 (50)
GIII	-		1/6 (16)		1/8 (12)		2/36 (6)
Stage							
Ta	1/9 (11)	0.19	-		1/45 (2)	0.93	3/357 (1)
T1	6/9 (67)	0.85	25/36(70)	0.99	31/45(69)	0.93	248/357 (70)
T2	1/9 (11)	0.77	4/36 (11)	0.81	5/45 (11)	0.74	30/357 (8)
T3	1/9 (11)	0.81	7/36 (19)	0.94	8/45 (18)	0.89	71/357 (20)
T4	-		-		-	-	5/357 (1)

* p-values are calculated with respect to carriers of noncarriers.

*GI-GIV–Fuhrman Grade.

**Fig 3:**
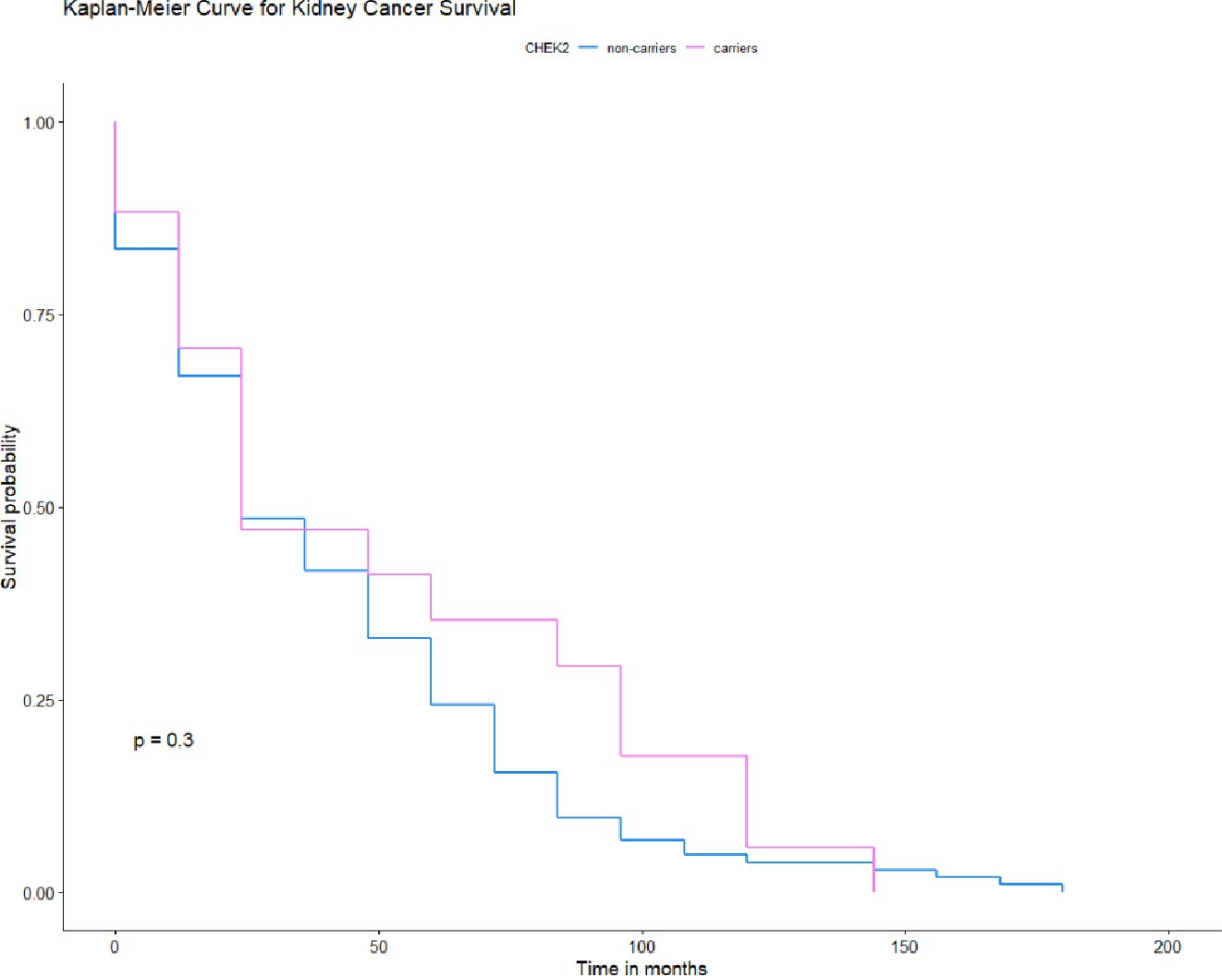
Kaplan-Meier survival curves of kidney cancer patients with any *CHEK2* mutation and -negative sub-cohorts.

None of the four variants in *CHEK2* appeared to influence the survival time of the patients with kidney cancer Fig 3 Nor did the have an effect on survival if the data was stratified for age, smoking status, cancer family history, sex and clinical characteristics. The median survival was the same for all *CHEK2* carriers was 24 months (Table 6). The 10-year survival was 5% for patients with truncation mutations and 38% for patients with missense mutations compared to 20% for non-carriers, after adjusting for age, smoking status, family history sex and clinical characteristics. The HR for mortality associated with kidney cancer and *CHEK2* mutation was 0.85 (95% CI 0.15–4.79; p = 0.9) for patients younger than 61 years old; 0.78 (95% CI 0.35–1.76; p = 0.5) for cases older than 61 years old; 0.19 (95% CI 0.02–1.49; p = 0.11) for non-smoking group; 0.93 (95% CI 0.40–2.19; p = 0.9) for smoking patients; 0.71 (95% CI 0.36–1.40; p = 0.3) for cases with no cancer family history; 0.62 (95% CI 0.16–2.36; p = 0.5) for females; 1.49 (95% CI 0.57–3.89; p = 0.4) for males; 0.68 (95% CI 0.25–1.84; p = 0.4) for patients with Stage T1; 1.90 (95% CI 0.35–10.5; p = 0.5) for patients with Stage T2; 1.29 (95% CI 0.28–5.93; p = 0.7) for patients with Stage T3; 0.60 (95% CI 0.28–1.29; *p* = 0.2) for patients with clear cell carcinoma and 1.32 (95% CI 0.22–8.01; *p* = 0.8) for patients with papillary kidney cancer. The Hazard ratio for patients with positive cancer family history, for patients with Stage, Ta, T4 and with chromophobe renal cell carcinoma could not be calculated due to the low number of patients in these groups.

**Table 6:** Survival of patients with kidney cancer; by variant alleles of *CHEK2*.

	Patients with truncation mutations (n = 9)	Patients with missense mutations (n = 36)	Patients with any *CHEK2* mutation (n = 45)	Patients with no mutation in *CHEK2* (n = 357)
Median follow-up (mo)	72	120	120	96
Proportion of deceased (%)	43	44	43	39
Median survival (mo)	24	24	24	24
5-Year survival (%)	24	42	45	58
10-Year survival (%)	5	38	34	20
HR	2.10	0.69	0.79	1.0
95% CI	0.49–8.96	0.35–1.39	0.42–1.51	-
p-value	0.3	0.3	0.5	-

Hazard ratio (HR), 95% confidence interval (CI), and p-values are calculated by coxph test. Data was stratified for age, sex, clinical characteristics, smoking status and cancer family history.

## Discussion

In this study, we found no impact of *CHEK2* mutations on bladder or kidney cancer survival. However, we observed a possible increased survival in the subgroup of patients with stage T1 bladder cancer with *CHEK2* mutations but it was not statistical significance, and more patients would need to be studied to draw conclusions. We noticed that *CHEK2* missense mutations were more common among patients with low grade invasive bladder cancer and in patients with stage Ta. Among patients with high grade invasive bladder cancer and in the group of patients with stage T1 bladder cancer the frequency of the I157T was lower. Our study revealed that any mutation in *CHEK2* occurs more often among patients with bladder cancer with stage Ta and stage T1 disease. Truncating *CHEK2* mutations has been associated with kidney cancer patients in the clear cell carcinoma subgroup of Fuhrman Grade II disease. Future studies are necessary to determine whether *CHEK2* mutations influence survival in patients with stage T1 disease. To our knowledge no such large study describing the clinical characteristics and survival of patients with bladder and kidney cancer and mutations in *CHEK2* has been reported to date.

Słojewski *et al*. suggested that mutations in *CHEK2* gene were a significant risk factor for the number of recurrences, characterized by a worse clinical course [14]. Their study lasted 2 years and included 24 patients with mutations in *CHEK2* and 44 controls. Patients of both groups were diagnosed with bladder cancer. Their main goal was to check the risk of recurrence rate and recurrence free survival among patients versus controls. Finally, they present ed a correlation between *CHEK2* mutations and the risk of recurrence but not with tumor grade. Spachmann *et al*. collected the group of 126 patients with urothelial bladder cancer in stage pT1 [15]. Their study ran for 8 years. They found that loss of *CHEK2* expression was associated with a worse progression-free survival, multifocal tumors, carcinoma in situ, and higher tumor grading (G3) [15]. Carlo *et al*. reported 254 cases with advanced renal cancer and showed a prognostic effect of *CHEK2* on urinary tract cancer predisposition [16]. Ge *et al*. examined three genome-wide association studies that included 3591 patients with bladder cancer and 1322 renal cell carcinoma patients and reported that the I157T variant found in *CHEK2* was associated with decreased risk of bladder cancer and renal cell carcinoma [17].

Based on a review of the evidence in the literature there are some studies that examine the impact of *CHEK2* mutations on patient survival who have been diagnosed with breast, prostate or pancreatic cancer. Huzarski *et al*. enrolled 3,592 women with breast cancer and the 10-year survival rates for all *CHEK2* mutation carriers and for non-carriers was similar. Only among women with ER-positive breast cancer did they find an adverse association between survival and the I157T *CHEK2* variant [10]. In a separate investigation, Muranen *et al*. included female breast cancer patients from 15 studies and they showed that the I157T variant of *CHEK 2* did not influence early death, breast cancer-specific survival or distant metastasis relapse between carriers and non-carriers. Moreover, the women with the 1100delC variant were characterized by a worse survival. However, the investigators did not find a difference in the analyses of subgroups of patients with ER-positive [18] disease. Weischer *et al*. enrolled 25,571 women with breast cancer from 22 studies and tested the 1100delC. variant. They showed that women with ER-positive breast cancer had a statistically significant greater risk of early death, breast cancer-specific death and of presenting with a second breast cancer [19]. Cybulski *et al*. examined 3750 men with prostate cancer and did not observe any difference in disease prognosis between carriers of *CHEK2* mutations and non-carriers [20]. Goldstein *et al*. observed that patients with pancreatic cancer and mutations in *CHEK2* gene and other genes responsible for DNA damage repair such as: *ATM*, *BRCA1/2*, *CDKN2A*, *ERCC4* and *PALB2* were characterized with better survival than patients without mutations [21]. However, this study has been performed on a small number of cases (n = 133).

Herein we found no impact of *CHEK2* mutations on survival from patients with cancer of bladder or kidney regardless of their age, smoking status, cancer family history and sex. Our results are consistent with the findings published by Cybulski et al. and Huzarski et al. pointing to there being no association between *CHEK2* mutation status and survival of breast and prostate cancer patients.

There are several strengths of our study including the number of patients with bladder and kidney cancer, which is complemented by the collection of detailed participant information. All patients were Polish. Predictors of include age, sex, cancer family history, clinical characteristics and smoking status were controlled for in our statistical approach. The maximum period of follow-up was 35 years.

In conclusion, this study reveals that CHEK2 does not appear to be associated with prognosis or grade of disease in either urothelial bladder cancer of kidney cancer. The data points towards other genetic factors being associated with these two malignancies and that more investigations are required to identify genetic factors that influence disease risk and or prognosis.

## References

[1] Cybulski C, Górski B, Huzarski T, Masojć B, Mierzejewski M, Debniak T, et al. *CHEK2* is a multiorgan cancer susceptibility gene. Am J Hum Genet. 2004 Dec;75(6):1131–5. doi: 10.1086/426403 Epub 2004 Oct 18. 15492928 PMC1182149

[2] Zlowocka-Perlowska Elzbieta, Narod Steven A, Cybulski Cezary. *CHEK2* Alleles Predispose to Renal Cancer in Poland Affiliations + expanddoi: 10.1001/jamaoncol.2019.0022 30816943

[3] Cybulski C, Wokołorczyk D, Huzarski T, Byrski T, Gronwald J, Górski B, et al. A deletion in *CHEK2* of 5,395 bp predisposes to breast cancer in Poland. Breast Cancer Res Treat. 2007 Mar;102(1):119–22. doi: 10.1007/s10549-006-9320-y Epub 2006 Aug 8. 16897426

[4] Złowocka E, Cybulski C, Górski B, Debniak T, Słojewski M, Wokołorczyk D, et al. Germline mutations in the *CHEK2* kinase gene are associated with an increased risk of bladder cancer. Int J Cancer. 2008 Feb 1;122(3):583–6. doi: 10.1002/ijc.23099 17918154

[5] Dong X, Wang L, Taniguchi K, Wang X, Cunningham JM, McDonnell SK, et al. Mutations in *CHEK2* associated with prostate cancer risk. Am J Hum Genet 2003;72:270–280. doi: 10.1086/346094 12533788 PMC379222

[6] CHEK2 Breast Cancer Case-Control Consortium (2004). *CHEK2**1100delC and susceptibility to breast cancer: a collaborative analysis involving 10,860 breast cancer cases and 9,065 controls from 10 studies. Am J Hum Genet 74:1175–1182. doi: 10.1086/421251 15122511 PMC1182081

[7] Cybulski C, Huzarski T, Górski B, Masojć B, Mierzejewski M, Dębniak T, et al. A novel founder *CHEK2* mutation is associated with increased prostate cancer risk. Cancer Res 2004;64:2677–2679. doi: 10.1158/0008-5472.can-04-0341 15087378

[8] Siołek M, Cybulski C, Gąsior-Perczak D, Kowalik A, Kozak-Klonowska B, Kowalska A, et al. *CHEK2* mutations and the risk of papillary thyroid cancer Int J Cancer. 2015 Aug 1;137(3):548–52. doi: 10.1002/ijc.29426 Epub 2015 Jan 28. 25583358

[9] Wasielewski M, Vasen H, Wijnen J, Hooning M, Dooijes D, Tops C, et al. *CHEK2* 1100delC is a susceptibility allele for HNPCC-related colorectal cancer. Clin Cancer Res. 2008 Aug 1;14(15):4989–94. doi: 10.1158/1078-0432.CCR-08-0389 18676774

[10] Huzarski T, Cybulski C, Wokolorczyk D, Jakubowska A, Byrski T, Gronwald J, et al. Survival from breast cancer in patients with *CHEK2* mutations. Breast Cancer Res Treat. 2014 Apr;144(2):397–403. doi: 10.1007/s10549-014-2865-2 Epub 2014 Feb 21. 24557336

[11] Teodorczyk U, Cybulski C, Wokołorczyk D, Jakubowska A, Starzyńska T, Lawniczak M, et al. The risk of gastric cancer in carriers of *CHEK2* mutations. Familial Cancer 2013;12(3):473–478. doi: 10.1007/s10689-012-9599-2 23296741

[12] Cybulski C, Wokołorczyk D, Kluźniak W, Jakubowska A, Górski B, Gronwald J, et al. An inherited NBN mutation is associated with poor prognosis prostate cancer. Br J Cancer 2012;13 doi: 10.1038/bjc.2012.486 23149842 PMC3566821

[13] Cybulski C, Wokołorczyk D, Jakubowska A, Huzarski T, Byrski T, Gronwald J, et al. Risk of breast cancer in women with a *CHEK2* mutation with and without a family history of breast cancer. J Clin Oncol 2011;29:3747–375. doi: 10.1200/JCO.2010.34.0778 21876083

[14] Słojewski M, Złowocka E, Cybulski C, Górski B, Debniak T, Wokołorczyk D, et al. *CHEK2* germline mutations correlate with recurrence rate in patients with superficial bladder cancer. Ann Acad Med Stetin. 2008;54(3):115–21. 19839522

[15] Spachmann PJ, Azzolina V, Weber F, Evert M, Eckstein M, Denzinger S, et al. Loss of *CHEK2* Predicts Progression in Stage pT1 Non-Muscle-Invasive Bladder Cancer (NMIBC). Pathol Oncol Res. 2020 Jul;26(3):1625–1632. doi: 10.1007/s12253-019-00745-7 Epub 2019 Sep 10. 31506803

[16] Carlo MI, Mukherjee S, Mandelker D, Vijai J, Kemel Y, Zhang L, et al. Prevalence of Germline Mutations in Cancer Susceptibility Genes in Patients With Advanced Renal Cell Carcinoma. 2018 Sep 1;4(9):1228–1235. 10.1001/jamaoncol.2018.1986 PMC6584283 29978187

[17] Ge Y, Wang Y, Shao W, Jin J, Du M, Ma G, et al. Rare variants in BRCA2 and *CHEK2* are associated withthe risk of urinary tract cancers. 2016; Sci Rep 6:33542. doi: 10.1038/srep33542 27632928 PMC5025839

[18] Muranen TA, Blomqvist C, Dörk T, Jakubowska A, Heikkilä P, Fagerholm R, et al. Patient survival and tumor characteristics associated with *CHEK2*:p.I157T - findings from the Breast Cancer Association Consortium. H. Breast Cancer Res. 2016 Oct 3;18(1):98. doi: 10.1186/s13058-016-0758-5 27716369 PMC5048645

[19] Weischer M, Nordestgaard BG, Pharoah P, Bolla MK, Nevanlinna H, J Van’t Veer L, et al. *CHEK2**1100delC heterozygosity in women with breast cancer associated with early death, breast cancer-specific death, and increased risk of a second breast cancer. J Clin Oncol 2012;30:4308–4316. doi: 10.1200/JCO.2012.42.7336 23109706 PMC3515767

[20] Cybulski C, Wokołorczyk D, Kluźniak W, Jakubowska A, Górski B, Gronwald J, et al. Polish Hereditary Prostate Cancer Consortium. An inherited NBN mutation is associated with poor prognosis prostate cancer. Br J Cancer. 2013 Feb 5;108(2):461–8. doi: 10.1038/bjc.2012.486 Epub 2012 Nov 13. 23149842 PMC3566821

[21] Goldstein JB, Zhao L, Wang X, Ghelman Y, Overman MJ, Javle MM, et al. Germline DNA Sequencing Reveals Novel Mutations Predictive of Overall Survival in a Cohort of Patients with Pancreatic Cancer. Clin Cancer Res. 2020 Mar 15;26(6):1385–1394. doi: 10.1158/1078-0432.CCR-19-0224 Epub 2019 Dec 23. 31871297

